# Purification, Characterization, and Self-Assembly of the Polysaccharide from *Allium schoenoprasum*

**DOI:** 10.3390/foods10061352

**Published:** 2021-06-11

**Authors:** Fengrui Zhang, Jun Zheng, Zeyu Li, Zixuan Cai, Fengqiao Wang, Dong Yang

**Affiliations:** 1 Beijing Key Laboratory of Functional Food from Plant Resources, College of Food Science & Nutritional Engineering, China Agricultural University, 17 East Tsinghua Rd., Beijing 100083, China; timzhang@alu.cau.edu.cn (F.Z.); zheng@cau.edu.cn (J.Z.); 2016309080315@cau.edu.cn (Z.L.); SY20183060967@cau.edu.cn (Z.C.); patrick0228@hotmail.com (F.W.); 2 Xinghua Industrial Research Centre for Food Science and Human Health, China Agricultural University, Xinghua 225700, China

**Keywords:** Chinese chive, *Allium schoenoprasum*, polysaccharide structure, hydrodynamic behavior

## Abstract

The major polysaccharide component from the stalk of *Allium schoenoprasum* (*As*sP) was extracted and purified. Gel filtration chromatography purified *As*sP exhibited a molecular weight of around 1.7 kDa, which was verified by MALDI-ToF-MS. The monosaccharide analysis revealed its composition as rhamnose: arabinose: galactose: glucose: mannose: fructose with a molar ratio of 0.03:2.46:3.71:3.35:1.00:9.93, respectively. The Congo-red assay indicated that there was no tertiary structure of this polysaccharide, however, it self-assembled into a homogenous nanoparticle with a diameter of ~600 nm as revealed by the dynamic light scattering measurement. The solution behavior of this polysaccharide was simulated. The association of this polysaccharide was both time dependent and concentration dependent. *As*sP forms spherical particles spontaneously as time passes by, and when the *As*sP concentration increased, the spherical particles increased their sizes and eventually merged into cylindrical micelles. The diversity of *As*sP hydrodynamic behavior endowed potential versatility in its future applications.

## 1. Introduction

*Allium schoenoprasum*, also known as Chinese chive, is a commonly seen vegetable and seasoning on the dining table worldwide since ancient times. It was shown to exhibit antioxidant activity, with the highest activity from its leaf of the cultivated plants and highest activity in its roots from the tissue culture plants [1,2,3]. The diallyl sulfide content in chive oil inhibits a wide spectrum of foodborne pathogens, while the phenolic compounds, steroidal saponins, exhibited antiproliferative effects [4,5]. Extracts from the chive leaves could inhibit Ehrlich carcinoma, and attenuates erythrocyte deformability in sickle cell anemia patients [6,7]. However, it was also found that cadmium and selenium could accumulate in this plant to a toxic level [8,9,10]. Thus, the study on the extracts from *Allium schoenoprasum,* which could efficiently eliminate heavy metal while maintain its biological function, become significant.

The stalk of the Chinese chive was shown to contain about 16% polysaccharides. The structure of this polysaccharides purified from the *Allium schoenoprasum* stalk (*As*sP) was preliminarily studied by Ying Zhang [11], and it showed an effect of reducing blood lipids of wistar rats [12]. More and more natural polysaccharides were investigated, such as these purified from *Penicillium griseofulvum*, *Dunaliella tertiolecta*, *Pleurotus ostreatus*, *Armillaria mellea*, and *Punica granatum*, and most of the studies were focusing on their biological functions while few reported their solution behaviors as encapsulating and delivering reagents [13,14,15,16,17]. Here, we purified the polysaccharides from *A. schoenoprasum*, further studied its structure from primary to tertiary with different conclusions as Zhang Ying previously reported, and its novel solution behavior both in the test tube and in silico. Results indicated this polysaccharide could potentially serve as a nanoparticle material in multiple circumstances.

## 2. Materials and Methods

### 2.1. Materials and Chemicals

Air dried *A. schoenoprasum* stalk was kindly offered by Jiangsu Natural Food Co., Ltd. (Xinghua, Jiangsu Province, China). Calcium chloride dehydrate, absolute ethanol, sulfuric acid, and aqueous ammonia of analytic grade were purchased from Beijing Chemical Works (Beijing, China).

### 2.2. Methods

#### 2.2.1. Extraction and Purification of *As*sP

Purification of chive polysaccharides was performed as the following. The ground, dry chive stalk was filtered through a 20 mesh screen, rinsed with 30-fold (by weight) of water for 2 h at 85 °C. The residue was removed with a gauze, and filtered to get a clear solution before being further concentrated with a rotary evaporator (RE-200, Yarong Biochemical Instrument, Shanghai, China). Addition of 4-fold (by volume) of anhydrous ethanol was performed to precipitate the polysaccharides overnight. After removal of the supernatant, the polysaccharide was resuspended in water and the ammonia solution was added to adjust the pH to 8.5. Then, the 10% calcium chloride solution was titrated (to remove pectin) until white precipitation appears in the solution. The solution was centrifuged to remove the pectin precipitation, and the titration was repeated until no more precipitation was seen. The solution was then loaded into a dialysis bag with a molecular cutoff of 500 Da, dialyzed against water 6 times at 4 °C for a total of 48 h. The *As*sP solution was then concentrated, filtered through a 0.22 μM filter, and purified on a HiPrep™ Sephacryl™ S-400 HR gel filtration chromatography column (GE Healthcare, Boston, MA, USA) to remove the impurities. The flow rate of water was set at 1 mL/min and every 5 mL of elution was collected. The polysaccharide content in each elution was monitored by measuring its absorbance at 200 nm on a DS-11 FX+ spectrophotometer (DeNovix, Wilmington, DE, USA). The polysaccharide concentration was measured with the phenol-sulfuric acid method, as previously described [18].

#### 2.2.2. Gel Permeation Chromatography

The purified *As*sP of 5 mg/mL was analyzed with a PL aquagel-OH Mixed-M GPC/SEC column (300 × 7.5 mm, Agilent, Santa Clara, CA, USA) on a 1260 Infinity II GPC/SEC system coupled with a G7800A refractive index detector. The mobile phase contains pure water which was set at a flow rate of 1.0 mL/min and a column temperature at 30 °C. The polysaccharides GPC standards analyzed in parallel are pullulan with molecular weights of 6600, 21,700, 113,000, 348,000 Da (ZZBio Co., LTD, Shanghai, China).

#### 2.2.3. Mass Spectroscopy

The *As*sP sample was mixed in an equal volume with 2,3-dihydroxybenzoic acid (DHB) and spotted onto a MALDI target pre-spotted with 5-chloro-2-mercaptobenzothiazole (CMBT). After air drying, crystallized spots were detected with a MALDI-TOF/TOF mass spectrometry (AB SCIEX 5800, AB Sciex, Framingham, MA, USA). Polysaccharide mass maps were acquired in the positive reflection mode with an accelerating voltage set at 20 kV. Spectra were obtained by accumulating 3500 laser shots for quantification with the laser intensity set at 6659. Calibration mixtures were used to calibrate the spectrum.

#### 2.2.4. Monosaccharide Composition Analysis

*As*sP was totally hydrolyzed with an equal volume of 0.4 mol/L trifluoroacetic acid (TFA) at 110 °C for 2 h in a sealed glass tube. The hydrolysate was dried under reduced pressure at a temperature lower than 40 °C. The same volume of methanol was added to the dry hydrolysate to dissolve residual TFA and evaporated under the above condition. This was repeated 3 times to scavenge TFA. The dried hydrolysate was dissolved in 1 mL of ultrapure water and stored at 4 °C for later use. The monosaccharide standard was made to 1 μg/mL solutions. Both the *As*sP hydrolysate and the monosaccharide standard were filtered with a 0.22 μm membrane filter before analyzed on a Dionex CarboPac PA10 analytical column (250 × 4 mm, Thermo Scientific, Waltham, MA, USA) installed on a Dionex ICS-5000^+^ ion chromatography system coupled with a DC detector (Thermo Scientific). The mobile phase contains a mixture of 80:20 (*v*:*v*) of water and 250 mM/L NaOH with a flowrate of 1.0 mL/min. The molar ratio of the monosaccharide component was calculated by area via the standard curve established with pure monosaccharide of known concentrations.

#### 2.2.5. UV-Vis Spectroscopy

The UV-Vis spectra of *As*sP (0.25 mg/mL) was recorded on a Hitachi U-3900H spectrophotometer (Hitachi, Tokyo, Japan) in a quartz cuvette with a light path of 1 cm. The absorbance was recorded between 190 and 800 nm with a step size of 1 nm.

#### 2.2.6. FT-IR Spectroscopy

FT-IR spectra of *As*sP was recorded on a PerkinElmer FT-IR spectrometer (Spectrum 100, PerkinElmer, Waltham, MA, USA). An aliquot of 5.0 mg freeze-dried *As*sP was blended with 200 mg potassium bromide into powder and pressed into pellets. The sample was scanned at room temperature under dry air, and 16 scans of the transmittance were recorded between 4000 and 452 cm^−1^ with a step size of 4 cm^−1^. The collected spectra was processed with the Spectrum 10 software (PerkinElmer).

#### 2.2.7. Dynamic Light Scattering

The particle size of the *As*sP was determined with a dynamic light scattering (DLS) instrument (Zetasizer Nano-ZS90, Malvern Instruments Ltd., Worcestershire, UK), and the measurement was carried out at 25 °C. The particle size was calculated by the Stokes-Einstein equation and reported as a cumulative mean diameter (size, nm) for size distribution [19].

#### 2.2.8. Congo-Red Test

The tertiary conformation of *As*sP was analyzed by the interaction with Congo-red. Briefly, 2 mg of the polysaccharide sample was mixed with 2 mL of distilled water and 2 mL of the Congo-red solution (80 μmol/L). Different volumes of the NaOH solution (1 mol/L) were gradually added to the mixture to obtain the final NaOH concentration of 0–0.5 mol/L. The mixed solution without polysaccharides was scanned as a control. The maximum absorption wavelength at different concentrations of the NaOH solution were determined in the range of 200–600 nm after equilibration at room temperature for 10 min [20].

#### 2.2.9. Computational Hydrodynamic Behavior Simulation

The dissipative particle dynamics (DPD), a mesoscopic scale simulation technique, was employed to simulate the hydrodynamic behavior of *As*sP molecules in an aqueous solution. Coarse-grained models were used to represent the *As*sP and water molecules. The Flory-Huggins parameters (*χ_ij_*) between component *i* and *j* were determined by approximation with solubility parameter *δ_i_* with the following formula [21]:

*χ_ij_* = *V(δ_i_ − δ_j_*)^2^/RT, (1)

where *V* is the arithmetic average of the molar volumes of components *i* and *j*. *δ_i_* and *δ_j_* are the solubility parameters of components *i* and *j*, respectively. R is the ideal gas constant and T (K) is the temperature of the system.

The solubility parameters of each component was calculated by the Amorphous Cell modules with the Materials Studio (BIOVIA, Dassault Systemes, Paris, France). The 3D structure of each component was constructed and energy minimized with the minimizer in the discover module. The *As*sP system was constructed with the construction function in the amorphous cell module, and energy minimized with the discover module. The dynamics function in the discover module was employed to calculate the system density which was then used to construct the *As*sP system again. Once the latest polysaccharide system was energy minimized, the final density was used to calculate the solubility parameter with the analysis in the amorphous cell module. Calculation of the repulsion parameters (*a_ij_*) between component *i* and *j* were determined by the Flory-Huggins parameter *χ_ij_* with the following formula [22]:

*a_ij_* = 25 + 3.27*χ_ij_*, *ρ* = 3, (2)

To perform the DPD simulation, a cubic simulation box with 30 × 30 × 30 R_c_^3^ side lengths was constructed. The default spring constant between the beads was set to 4.0, and the time step was set at 0.05. The DPD simulation was carried out with the DPD module implemented in MS.

## 3. Results

### 3.1. Extraction, Purification, and Molecular Weight Characterization of AssP

**Figure 1:**
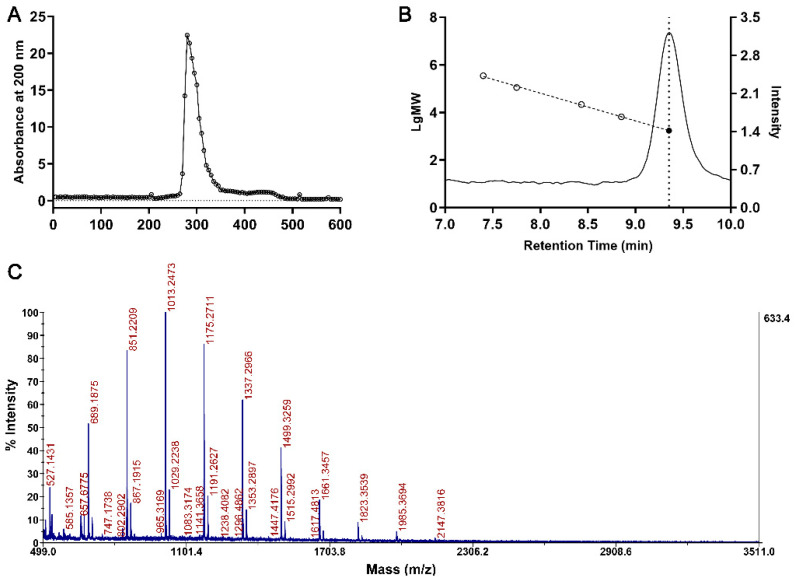
Purification and molecular weight characterization of *As*sP. (**A**) The extracted *As*sP was concentrated and the impurities were removed with a S-400 HR gel filtration chromatography column. Each 5 mL of elution was collected, and the content of total carbohydrates in each fraction was monitored by its absorbance at 200 nm. (**B**) Gel permeation chromatogram of the polysaccharide standards (not shown) and purified *As*sP (right *y* axis). The retention time and log value of molecular standards (empty circles) regressed into a linear relationship with the R^2^ value of 0.99 (left axis). The retention time of *As*sP (dotted line vertical to the *x*-axis) corresponds to a molecule weight of 1722 Da (filled dot, intercept with the standard curve). (**C**) MALDI-TOF/TOF mass spectrum of purified *As*sP.

Hot water was used to extract the polysaccharide from the stalk of the Chinese chive, and crude polysaccharide was ethanol precipitated for its safety and simplicity for medicine and food products [23]. After pectin removal by CaCl_2_ titration, the polysaccharide was dialyzed to remove small molecules. The polysaccharide was further purified with a gel filtration chromatograph, and each fraction of elution was detected. In our experimental setup, *As*sP eluted at around 270–330 mL with little impurities (Figure 1A). The extraction yield is ~2.95% by weight.

Multiple methods were employed to measure the molecular weight of *As*sP, including GPC and MALDI-TOF/TOF mass spectrometry. Firstly, the polysaccharide molecular standard of pullulan with a variety of molecular weight (6600, 21,700, 113,000, 348,000 Da) was loaded to a GPC column and each elution time was obtained. The log value of their molecular weight was plotted against each corresponding elution time and a standard curve with R^2^ of 0.99 was obtained. The purified *As*sP was analyzed with identical procedures, and its elution peak corresponding to a time range of 9.1–9.8 min centered at 9.35 min (Figure 1B). This corresponded to a molecular weight range of 3363–516 Da centered around 1722 Da. The calculated Mn of *As*sP is 1512 Da, and Mw of 1892 Da with PDI of 1.25. To further identify the molecular weight of *As*sP, MALDI-TOF/TOF was employed to measure its accurate molecular weight. A cluster of peaks with 162 Da differences appeared on the mass spectrum in a range of 527.14–2147.38 Da, which is consistent with the GPC analysis (Figure 1C).

### 3.2. Structural Characterization of AssP

**Figure 2:**
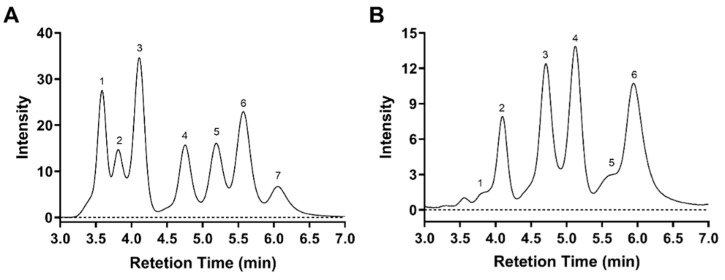
Monosaccharide composition of *As*sP. (**A**) Chromatogram of monosaccharide standard: 1, galactosamine; 2, rhamnose; 3, arabinose; 4, galactose; 5, glucose; 6, mannose; 7, fructose. (**B**) Chromatogram of *As*sp with peak identity: 1, rhamnose; 2, arabinose; 3, galactose; 4, glucose; 5, mannose; 6, fructose.

To determine the monosaccharide composition of *As*sP, the polysaccharide was acid hydrolyzed before the ion chromatographic analysis. Monosaccharide standards of galactosamine, rhamnose, arabinose, galactose, glucose, mannose, and fructose were analyzed in parallel to establish the curve for the calculation of each mole fraction based on previous research [24]. The chromatogram of each of the above monosaccharide exhibited a peak area that yielded a linear relationship with their concentrations, which enabled us to calculate the corresponding mole fraction (Figure 2A, Figure S1, Table S1). The molar ratios of monosaccharides were calculated as 0.03:2.46:3.71:3.35:1.00:9.93 of rhamnose: arabinose: galactose: glucose: mannose: fructose in the purified *As*sP (Figure 2B). It is worthy to mention that in the previous study [24], the ratio of 5.1:22.6:31.5:1 as arabinose: galactose: glucose: mannose was obtained for *As*sP by Ying Zhang et al. This is probably due to the limitation of the 1-phenyl-3-methyl-5-pyrazolone (PMP)-derivation method they used for the monosaccharide analysis, which could not detect the presence of fructose.

**Figure 3:**
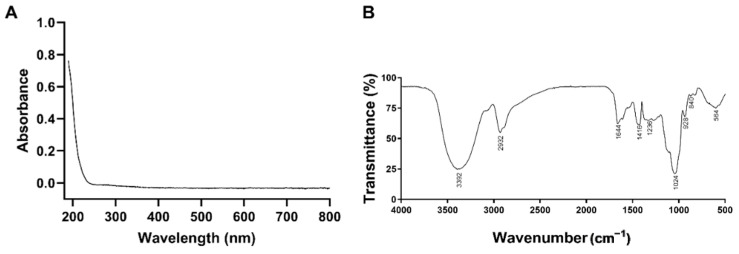
UV-Vis and FT-IR spectrums of *As*sP. (**A**)The UV-Vis spectrum of an *As*sP water solution from 190–800 nm. (**B**) The transmittance of *As*sP mixed with KBr on a FT-IR spectrometer of 500–4000 cm^−1^.

The purified *As*sP was subjected to the UV-Vis analysis. As shown in Figure 3A, the UV-Vis spectrum of gel filtration purified *As*sP yielded a significant absorption at 192 nm while no absorbance at 260 or 280 nm, indicating non-detectable nucleotide or protein in the purified *As*sP [25]. As shown in Figure 3B, functional groups of the *As*sP (the KBr background deducted) were characterized with FT-IR. The strong and broad bands at ~3392 cm^−1^ indicates the hydroxyl (O−H) stretching vibration of the polysaccharide chain. Bands at 2932 cm^−1^ were ascribed to the asymmetric C−H stretching vibration. Absorption at 1644 cm^−1^ was assigned to the carbonyl stretching of the polysaccharide. The peak at 1416 cm^−1^ corresponded to the C=O stretching and C−O bond from the carboxyl group and C−H bending vibrations. The band at 1024 cm^−1^ corresponded to the C−OH bonds, and the band at 928 cm^−1^ corresponded to the asymmetric stretching of pyranose ring in the polysaccharide chain.

### 3.3. Tertiary Structure and Solution Behavior of AssP

**Figure 4:**
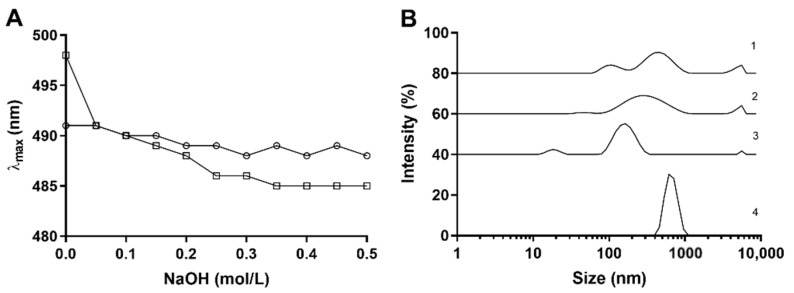
Tertiary structure characterization of *As*sP. (**A**) The maximum absorbance wavelength of Congo-red (square) and Congo-red *As*sP mixture (circle) solution as the NaOH concentration changes. (**B**) The dynamic light scattering intensity of *As*sP solution as a function of the cumulative mean diameter at each purification step: 1, water extraction; 2, after ethanol precipitation; 3, after pectin removal; 4, after gel filtration purification.

Polysaccharides containing triple-helical structures form a Congo-red-polysaccharide complex, and the maximum absorption wavelength would have red-shifted compared with the Congo-red solution. The addition of strong alkali disrupts the hydrogen bonding thus the red-shift of the Congo-red-polysaccharide complex is weakened [18]. The tertiary structure of purified *As*sP was examined with the Congo-red assay as shown in Figure 4A. The maximum absorption wavelength of the *As*sP-Congo-red mixture did not yield any dramatic change (491 to 489 nm) as the concentration of NaOH increased, indicating the absence of triple-helical structure of *As*sP in the solution.

The polysaccharide solution of each purification step was examined with DLS to reveal the solution behavior of *As*sP at each stage (Figure 4B). Water extraction of the Chinese chive yielded a mixture of multiple-size polysaccharide solution, with the particle size ranging from 100 to 5000 nm. After precipitation with ethanol, the large proportion of polysaccharide with the size ranging from 100 to 500 nm were homogenized to ~300 nm, while the largest particle at ~5000 nm did not change. The removal of pectin further homogenized the polysaccharide solution to a ~150 nm size particle solution as indicated by the narrower peak. Notably, gel filtration which further purified *As*sP rendered this polysaccharide to form a more homogenous, but larger size (with an average size of ~600 nm) particle in the solution. This indicated a great potential for *As*sP to be used as a natural encapsulating/delivering reagent in the future.

**Figure 5:**
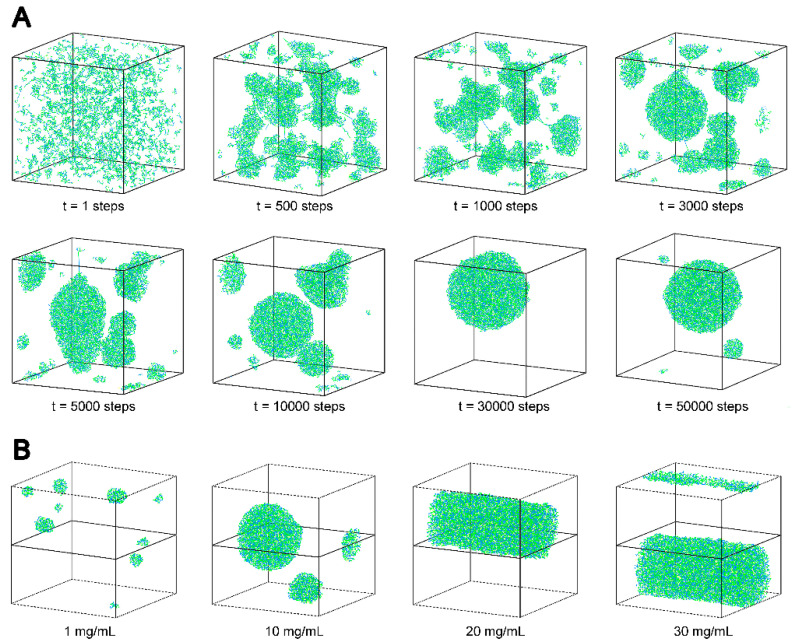
Simulation of the solution behavior of *As*sP. (**A**) The simulation of solution behavior of *As*sP molecules over time. (**B**) The simulation of the equilibrium solution state of *As*sP molecules at different concentrations.

To visualize the scenario of *As*sP assembly, its solution state was simulated with sugar residues represented by coarse-grained bead to calculate their interactions (Figure S2, Table S2). This method does not depend on the exact glycosidic linkages between the coarse-grained monosaccharide, but emphasized on the solution distribution and particle formation (Figure 5A). At the very beginning of time (t = 1 step), the *As*sP molecules distributed homogenously in the aqueous solution. As time passed (t = 500 steps), the polysaccharide molecules assembled into irregularly sized particles. Large spherical particles began to show up at a time point (t = 3000 steps), and the system tended to favor this type of solution behavior where eventually all the *As*sP molecules are distributed in the uniformly sized spherical particles (t = 30,000 steps). This is so far consistent with our DLS study (Figure 5A). However, it is likely a metastable state. At an approximate infinite time point (t = 50,000 steps), the *As*sP spherical particles began to disassemble into differently sized particles, suggesting an eventually unstable assembly of this polysaccharide nanoparticle.

The solution particle was also concentration dependent as the equilibrium state (t = 50,000 steps) of different concentrations of *As*sP were simulated (Figure 5B). At low concentrations, such as 1 mg/mL, the polysaccharide molecules form small spherical particles. As the concentration increased (10 mg/mL), the small particles approached each other and merged into larger particles. The spherical particles would merge into cylindrical micelle at higher *As*sP concentrations (20 mg/mL), and the diameter of the cylinder would increase as the concentration further increased (30 mg/mL). The diversity of the *As*sP solution particle forms suggested a strong versatility in the application.

## 4. Discussion

Hot water extraction of polysaccharides from the terrestrial plant has been the most efficient, low cost, and environmental-friendly method in a variety of applications [26,27]. One of the main purposes of this study is to remove the potential heavy metal accumulation in this vegetable, thus careful use of edible chemical reagents were paid special attention through-out the purification steps. Size exclusion chromatography identified a single peak after the extraction steps such as water extraction, evaporation/concentration, ethanol precipitation, pH adjustment, calcium precipitation, and dialysis. This comparatively simple set of purification steps suggests the potential feasibility in industrial purification of *As*sP.

The molecular weight and monosaccharide composition of *As*sP were identified. Unlike these more abundant polysaccharides from terrestrial biomass (e.g., xylans or cellulose) which has much larger molecular weights, *As*sP is composed of only ~10 sugar residues [28,29]. This of course contributes to the high solubility of *As*sP in water. What is more, the monosaccharide composition in *As*sP is more even. Although there is no tertiary structure in *As*sP, DLS experiments indicate that *As*sP eventually self-assembled into homogenous nanoparticles with a diameter of ~600 nm in the solution. The particle size is not static but constantly changing during the purification steps, suggesting a concentration and time dependent solution behavior of *As*sP. More interestingly, *As*sP forms spherical nanoparticles at a concentration of 10 mg/mL and lower concentrations or micelles at a concentration of 20 mg/mL and higher concentrations. This phenomenon of concentration dependent nanoparticle formation was reported with polyelectrolyte complex dispersions (PECs) formed nanoparticles [30]. However, it is rarely seen in biomass-based nanoparticles.

Biopolymeric nanoparticles are often categorized into polysaccharide nanoparticles and protein nanoparticles, which all find applications in drug delivery in a controlled or sustained or targeted manner [31]. Nanoparticles made from carbohydrates or polysaccharide played a wide role in the pharmaceutical industry due to their excellent biocompatibility, which are often used to deliver proteins, peptides, and nucleic acids [32,33,34]. Fabrication of biomass-based nanoparticles often involves a variety of techniques including desolvation, coacervation, emulsification-diffusion, and electrohydrodynamic atomization. *As*sP could self-assemble into either spherical nanoparticles or micelles at different diameters with a fine tune of solution concentrations without the above-mentioned fabrication processes. This is, to the best of our knowledge, not reported previously and suggest a promising application of *As*sP as a self-assembly biomaterial of nanoparticles for different scenarios.

## 5. Conclusions

The major polysaccharide component of a tradition vegetable *Allium schoenoprasum* was purified and characterized. Different from the previous report, our study revealed that a monosaccharide composition of this *As*sP was arabinose: galactose: glucose: fructose of approximately 1:2:2:5. This polysaccharide forms ~600 nm nanoparticles in the water solution and exhibited both a time dependent and concentration dependent behavior in forming different solution assemblies. This behavior diversity could serve in different future applications in ingredients encapsulation and delivery.

## Data Availability

Data are contained within the article or Supplementary Materials.

## Supplementary Materials

The following are available online at https://www.mdpi.com/article/10.3390/foods10061352/s1. Figure S1: The fitting curve of monosaccharide standard with the HPLC peak area; Table S1: Fitting curve parameters of monosaccharide standards; Figure S2: Coarse-grained model structure of *As*sP in the aqueous solution; Table S2: Repulsion parameters between monosaccharide residue beads.
