# Supplementary material for: Purification, Characterization, and Self-Assembly of the Polysaccharide from Allium schoenoprasum
Source: Foods. 2021 Jun 11;10(6):1352. doi: 10.3390/foods10061352 (PMC8230776; doi:10.3390/foods10061352)
Supplement: Supplementary file 1 [file foods-10-01352-s001.zip › foods-1205369-supplementary.pdf]

Supplementary information

Purification, characterization, and self-assembly of the polysaccharides from *Allium schoenoprasum*

Fengrui Zhang[1], Jun Zheng[1], Zeyu Li[1], Zixuan Cai[1,2], Fengqiao Wang[1], and Dong Yang[1,*]

[1] Beijing Key Laboratory of Functional Food from Plant Resources, College of Food Science & Nutritional Engineering, China Agricultural University, 17 East Tsinghua Rd., Beijing 100083, China
[2] Xinghua Industrial Research Centre for Food Science and Human Health, China Agricultural University, Xinghua 225700, Jiangsu Province, China
* Correspondence: dyang@cau.edu.cn; Tel.: +86-0106-273-7129

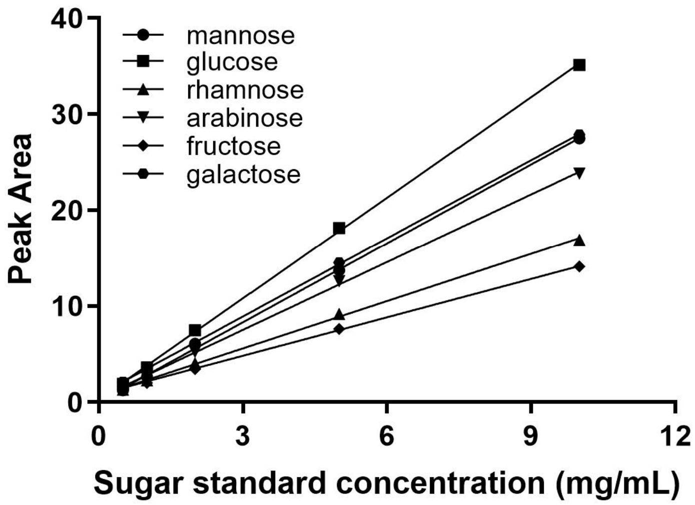

Figure S1. The fitting curve of monosaccharide standard with HPLC peak area. The peak area of standard monosaccharide solutions with known concentrations were plotted against their corresponding concentrations to yield fitting curves for monosaccharide composition analysis.

Table S1. Fitting curve parameters of monosaccharide standards.

Sugar Standard	Fitting Curve	R^2
rhamnose	y = 1.6409x + 0.6746	0.9990
arabinose	y = 2.3535x + 0.4621	0.9995
galactose	y = 2.7162x + 0.768	0.9996
glucose	y = 3.4976x + 0.305	0.9997
mannose	y = 2.7423x + 0.0856	0.9993
fructose	y = 1.3325x + 0.8339	0.9995

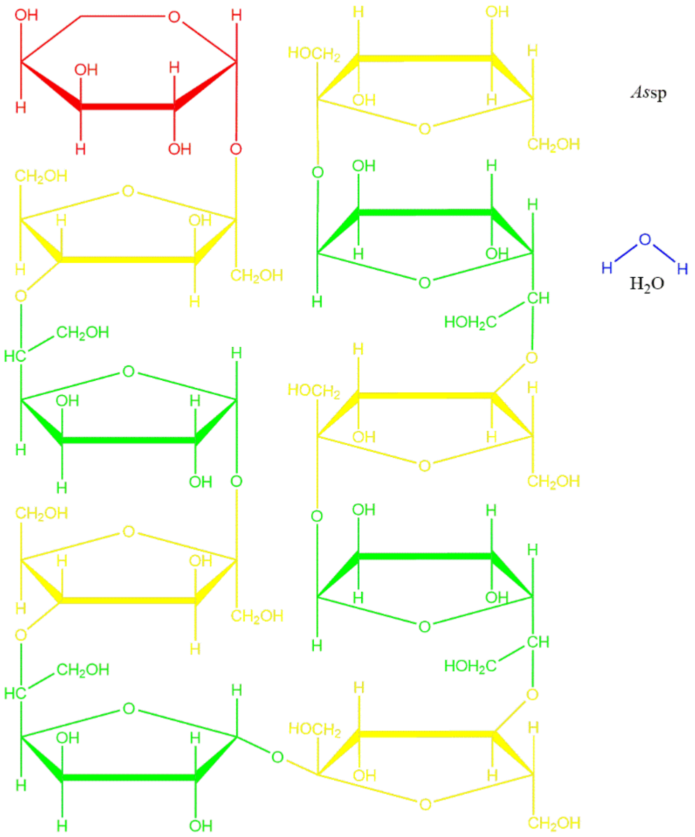

Figure S2. Coarse-grained model structure of *As*sP in aqueous solution. The coarse-grained model of the polysaccharide *As*sP and water molecules in an aqueous solution are represented by different beads. Red, arabinose residues; yellow, fructose residues; green, glucose or galactose residues; blue, water molecules.

Table S2. Repulsion parameters between monosaccharide residue beads.

Bead	W	A	F	G
W	25.000	-	-	-
A	88.586	25.000	-	-
F	83.606	25.505	25.000	-
G	92.185	25.037	25.286	25.000

A, arabinose residue; F, fructose residue; G, glucose or galactose residue; W, water.
